# 

**Published:** 1893-09

**Authors:** 


### CONTENTS.

COMMUNICATIONS.

Experiments with Bichloride of Mercury ........ 417
Charity ........ 422
Primary Causes of Dental Caries ........ 426

PROCEEDINGS.

Georgia State Dental Society ........ 427
Vermont State Dental Society ........ 437
The American Dental Association ........ 439
The National Association of Dental Faculties ........ 441
National Association of Dental Examiners ........ 444
Frontal Headache and Iodide of Potash ........ 446

SELECTIONS.

Abstract of a Lecture on "How can Diseases of the Nose and Throat Affect the Teeth?" ........ 447
Japanese Nu sery Notes ........ 457
The Necessity for Trained and Educated Health Officials ........ 458
Some Erroneous Ideas of Ptomaines ........ 459
Medical Hints ........ 461
New Method of Penetrating the Antrum of Highmore ........ 462
Do the Sick ever Sneeze? ........ 463
Toothache ........ 464
Vegetable Essences as Antiseptic Dressings ........ 464
How to Rest ........ 465

EDITORIAL.

The World's Columbian Congress ........ 465

